# Viral Infection Profile in Children Treated for Acute Lymphoblastic Leukemia—Results of Nationwide Study

**DOI:** 10.3390/pathogens11101091

**Published:** 2022-09-24

**Authors:** Joanna Zawitkowska, Katarzyna Drabko, Krzysztof Czyżewski, Magdalena Dziedzic, Kamila Jaremek, Patrycja Zalas-Więcek, Anna Szmydki-Baran, Łukasz Hutnik, Michał Matysiak, Wojciech Czogała, Walentyna Balwierz, Iwona Żak, Małgorzata Salamonowicz-Bodzioch, Bernarda Kazanowska, Grażyna Wróbel, Krzysztof Kałwak, Renata Tomaszewska, Tomasz Szczepański, Olga Zając-Spychała, Jacek Wachowiak, Marcin Płonowski, Maryna Krawczuk-Rybak, Aleksandra Królak, Tomasz Ociepa, Tomasz Urasiński, Filip Pierlejewski, Wojciech Młynarski, Justyna Urbańska-Rakus, Katarzyna Machnik, Sonia Pająk, Wanda Badowska, Tomasz Brzeski, Katarzyna Mycko, Hanna Mańko-Glińska, Agnieszka Urbanek-Dądela, Grażyna Karolczyk, Agnieszka Mizia-Malarz, Weronika Stolpa, Katarzyna Skowron-Kandzia, Jakub Musiał, Radosław Chaber, Ninela Irga-Jaworska, Ewa Bień, Jan Styczyński

**Affiliations:** 1 Department of Pediatric Hematology, Oncology and Transplantation, Medical University, 20-059 Lublin, Poland; 2 Department of Pediatric Hematology and Oncology, Collegium Medicum in Bydgoszcz, Nicolaus Copernicus University in Torun, 85-094 Bydgoszcz, Poland; 3 Department of Microbiology, Collegium Medicum in Bydgoszcz, Nicolaus Copernicus University in Torun, 85-094 Bydgoszcz, Poland; 4 Department of Hematology and Pediatrics, Medical University of Warsaw, 02-091 Warszawa, Poland; 5 Department of Pediatric Oncology and Hematology, Institute of Pediatrics, Jagiellonian University Medical College, 31-008 Krakow, Poland; 6 Department of Microbiology, University Children’s Hospital, Jagiellonian University Medical College, 31-008 Krakow, Poland; 7 Department of Paediatric Bone Marrow Transplantation, Oncology and Haematology, Wroclaw Medical University, 50-367 Wroclaw, Poland; 8 Department of Pediatrics, Hematology and Oncology, Medical University of Silesia, 40-752 Katowice, Poland; 9 Department of Pediatric Oncology, Hematology and Transplantology, Poznan University of Medical Sciences, 61-701 Poznan, Poland; 10 Department of Pediatric Oncology, Hematology, Medical University of Bialystok, 15-089 Bialystok, Poland; 11 Department of Pediatrics, Hemato-Oncology and Gastroenterology, Pomeranian Medical University, 70-204 Szczecin, Poland; 12 Department of Pediatrics, Oncology & Hematology, Medical University of Lodz, 90-647 Lodz, Poland; 13 Unit of Pediatric Hematology and Oncology, City Hospital, 41-500 Chorzow, Poland; 14 Clinical Department of Pediatric Oncology and Hematology, Department of Clinical Pediatrics, University of Warmia and Mazury in Olsztyn, Regional Specialized Children’s Hospital in Olsztyn, 10-561 Olsztyn, Poland; 15 Department of Pediatric Oncology and Hematology, Collegium Medium of Jan Kochanowski University in Kielce, 25-317 Kielce, Poland; 16 Department of Oncology, Hematology and Chemotherapy, Upper Silesia Children’s Care Health, Medical University of Silesia, 40-752 Katowice, Poland; 17 Clinic of Paediatric Oncology and Haematology, Faculty of Medicine, University of Rzeszow, 35-959 Rzeszow, Poland; 18 Department of Pediatrics, Hematology, Oncology and Endocrinology, Medical University of Gdansk, 80-210 Gdansk, Poland

**Keywords:** viral infection, acute lymphoblastic leukemia, chemotherapy, children

## Abstract

Viral infections can be a serious complication of therapy in children with acute lymphoblastic leukemia (ALL). In this study, we focused on the incidence and the profile of viral infection in children with ALL treated in 17 pediatric oncology centers in Poland in the two-year periods of 2018–2019 and 2020–2021. We also compared the frequency of viral infections in 2018–2019 to that in 2020–2021. In 2020–2021, a total of 192 children with ALL had a viral infection during intensive chemotherapy. A total number of 312 episodes of viral infections were diagnosed. The most common infections detected in the samples were: COVID-19 (23%), rhinovirus (18%), and respiratory syncytial virus (14%). COVID-19 and BK virus infections were the reason for the death 1% of all patients. In 2018–2019, a total of 53 ALL patients who had a viral infection were reported and 72 viral events were observed, mainly adenovirus (48.6%), rotavirus (31.9%), and herpes zoster (8.3%). No deaths were reported during this period. The cumulative incidence of viral infections in 2018–2019 was 10.4%, while for 2020–2021, it was 36.7%. In conclusion, a high incidence of COVID-19 infection was observed among pediatric patients with ALL in Poland. The mortality rate in our material was low. The viral profile in ALL children undergoing chemotherapy can be useful for clinicians to improve prophylactic and therapeutic strategies.

## 1. Introduction

Viral infections can be a serious complication of therapy in children with acute lymphoblastic leukemia (ALL). There are many factors such as the disease itself, intensive chemotherapy and steroid therapy, and prolonged neutropenia that make ALL pediatric patients vulnerable to them [1]. Additionally, coronavirus disease 2019 (COVID-19) caused by the novel severe acute respiratory syndrome coronavirus 2 (SARS-CoV-2) β-coronavirus has increased the risk of mortality in children with malignancies [2]. Patients who tested positive were either isolated at home or were directed to specialized units of infectious diseases if they were symptomatic or required medical treatment [2,3]. The European Centre for Disease Prevention and Control (ECDC) developed guidelines for COVID-19 isolation that were issued September 2, 2020 [4]. New therapeutic approaches such as monoclonal antibodies in childhood ALL can result in an improved prognosis, but on the other hand, can also increase susceptibility to viral infections [1]. Therefore, the prevention and improvement of supportive care, as well as the assessment of the incidence of infectious complications, are of great importance for pediatric patients with ALL [1,5,6].

The aim of the study was to analyze the incidence and the profile of viral infection in 1021 children with newly diagnosed acute lymphoblastic leukemia treated in 17 pediatric oncology centers in Poland in the two-year periods of 2018–2019 and 2020–2021. Additionally, we also compared the frequency of viral infections in 2018–2019 to that in 2020–2021.

## 2. Materials and Methods

Children with ALL received conventional chemotherapy in accordance with the therapeutic protocol in force in Poland. The study was approved by the Ethics Committee of Collegium Medicum in Bydgoszcz, Nicolaus Copernicus University in Torun, Poland.

### 2.1. Definition

Testing for viruses was conducted in ALL patients with clinical symptoms of infection. Viral infections were described when the virus was confirmed by the polymerase chain reaction (PCR) method in material derived from blood (cytomegalovirus, CMV; Epstein–Barr virus, EBV; adenovirus, ADV; coronaviruses OC43 and HKU1; and parvovirus B19), urine (BKV or ADV), stool (ADV, rotavirus, norovirus, sapovirus, or astrovirus) or cerebrospinal fluid (CMV, EBV, or HHV6). Respiratory syncytial virus (RSV), parainfluenza virus (PIF), influenza (FLU), metapneumovirus (MPV), rhinovirus (HRV), bocavirus (HboV) were detected using PCR from respiratory swabs or bronchoalveolar lavage. In all pediatric oncology centers in Poland, the detection of respiratory viruses is conducted using multiplex PCR, while singleplex PCR is used for other viral infections such as CMV, EBV, BKV, and ADV. Nasopharynx swabs were used as the material for virus testing, and PCR tests were performed to diagnose SARS-CoV-2 replication [3,7,8,9]. 

Viral infections were identified as sporadic and latent. The following sporadic viral infections were analyzed: influenza, PIF, MPV, RSV, ADV, coronaviruses, HRV, rotavirus, norovirus, sapovirus, astroviruses, poxvirus, and bocavirus. Many viruses such as EBV, BKV, VZV, HHV6, HSV, and CMV tend to cause latent infections. Herpesviruses infections were reported when clinical symptoms occurred (HSV or VZV) or viremia was diagnosed by PCR (CMV, EBV, or HHV6) [7,10]. 

### 2.2. Supportive Care

Supportive therapy was administered in accordance with institutional standards and treatment protocol recommendations. Children undergoing chemotherapy received oral co-trimoxazole and oral fluconazole. Oral acyclovir was used in patients who were in contact with persons sustaining varicella or a zoster infection and HSV. Additionally, the recombinant human granulocyte colony-stimulating factor (Rh-G-CSF) was managed during sepsis in the neutropenic phase or in high-risk pediatric patients. Preparations of immunoglobulin were used in the case of a decreased immunoglobulin concentration during infection in neutropenic patients. Children in the period of neutropenia were on a low-bacterial diet and kept under strengthened hygiene processes. There was no specific BKV prophylaxis; patients were monitored weekly [7,11].

Patients with a SARS-CoV-2 infection were separated; their isolation times depended on applicable laws in Poland that have changed during the time of the pandemic [3]. Additionally, Poland adopted the European Centre for Disease Prevention and Control (ECDC) guidelines on the ending of COVID-19 isolation [4]. Additionally, education and vaccination of all family members and healthcare professionals working with immunocompromised patients were very important [9].

### 2.3. Statistical Analysis

An analysis was conducted in R statistical software (version 4.2.2); α = 0.05. Respondents were divided into two groups: patients with one infection and patients with more than one infection. Dependencies between groups and other qualitative variables were analyzed using a chi-squared test or Fisher’s exact test. Quantitative variables were compared between two groups using a Mann–Whitney U test. Frequencies for all infections as well as the percentage of patients that died due to the infections are shown in the tables below. Some analyses were done using the number of infections observed in the sample (n = 312) and some using the number of patients (n = 192). Additionally, Kaplan–Meier survival curves for the cumulative incidence of viral infections were prepared and included a comparison between the periods of 2018–2019 and 2020–2021 based on a chi-squared log-rank test.

## 3. Results

For 2020–2021, the analysis included 510 children with initial ALL aged 1 to 18 years (median 4.62 years) at the time of diagnosis. A total number of 192/510 (37.5%) children experienced a viral infection during therapy for ALL. Among the analyzed group, 312 episodes of viral infections were diagnosed and ranged from 1 to 5 per person. The most common infections detected in the samples were as follows: COVID-19 infection (23%), rhinovirus infection (18%), and respiratory syncytial virus (RSV) infection (14%) (Table 1).

Most of the patients (63.5%) had one episode of a viral infection. Characteristics of patients who had reported 1 and >1 episode are presented in Table 1. Patients with one or more than one event of viral infection did not differ in the proportion of females in both groups. Both groups did not differ in time from age of patient to date of ALL diagnosis. Two patients died due to these infections (1% of total group). There was no significant correlation between death and the number of infections (*p* > 0.999). Both patients died due to a COVID-19 infection, but one of those patients also had a BKV (BK virus) infection. The time from diagnosis to death for a patient who died due to COVID-19 only was 53 days; for the second patient, the time from diagnosis of BKV to death was 14 days, while that from diagnosis of COVID-19 to death was 51 days.

In 2018–2019, a total number of 53/511 (10.4%) of the ALL patients were reported to have a viral infection during chemotherapy. When analyzing this group, 72 viral infection events were observed, mainly ADV (48.6%), rotavirus (31.9%), and herpes zoster (8.3%). No deaths were reported during this period. The comparison of 2018–2019 and 2020–2021 revealed significant differences in the frequency of different types of viruses (Table 2).

**Figure 1:**
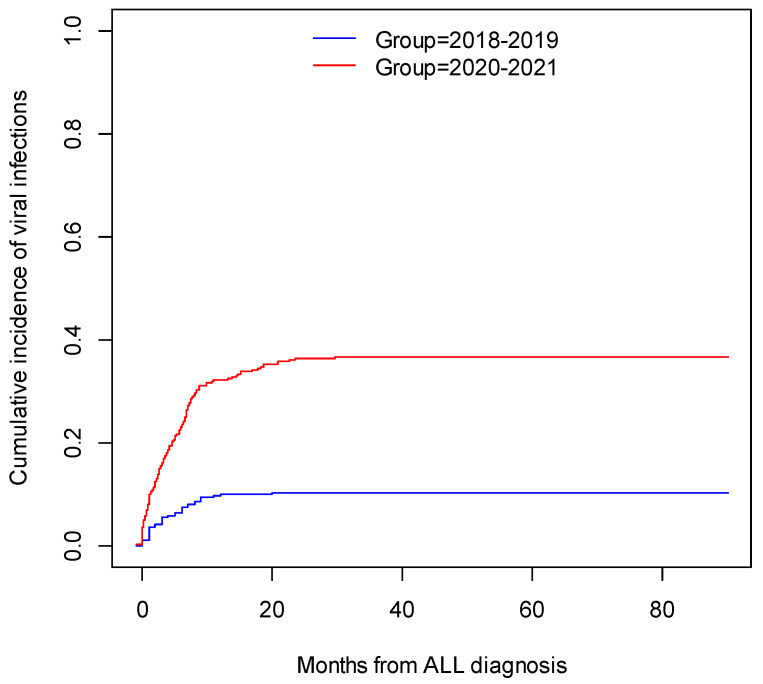
The cumulative incidence of viral infections in patients with ALL.

The cumulative incidence of viral infections in 2018–2019 (53 patients, 72 infections) was 10.4% CI95 [7.7%; 13.0%], while for 2020–2021 (192 patients, 312 infections), it was 36.7% CI95 [32.4%; 40.7%]; *p* (log-rank) < 0.001. The cumulative incidence of viral infections is illustrated in Figure 1. 

## 4. Discussion

Currently, the overall survival of children with ALL has improved, with Jeha’s recent trial demonstrating 96% [12]. This approach was made possible by several factors such as new diagnostic and therapeutic methods, the evaluation of minimal residual disease, and supportive care. However, the toxicities of chemotherapy, infections leading to the interruption of chemotherapy, and prolonged hospitalization are still the causes of death in children with ALL [13,14,15,16].

O’Connor et al. reported 75 patients with ALL and infection-related mortality (IRM) in the United Kingdom Childhood Acute Lymphoblastic Leukaemia Randomised Trial 2003 (UKALL 2003). In this study, the 5-year cumulative incidence of IRM was 2.4% (95% confidence interval (CI): 1.9–3.0%), accounting for 75 (30%) of 249 deaths. Seven (12%) children died due to the viral infection (adenovirus 3/43%, RSV 2/28%, and VZV 2/29%) [17].

In the Inaba trial, the cumulative risk of infection-related mortality was low (1.0%) compared to other studies (1.7–2.4%), although different therapeutic protocols made it difficult to directly compare [5,6,18].

Katsimpardi et al. described 58 pediatric cases (9.5% of the total) of specific viral infections. Of those, varicella-zoster virus (VZV) infections were the most common diagnosis (26 cases, 45%) followed by HSV infection (25 cases, 43%). Coxsackie virus, Epstein–Barr virus, and mumps virus were confirmed in two cases each (3% each), and one case of parvovirus B19 infection (2%) was diagnosed. No patient died due to a viral infection [19].

In our previous study published in 2019, we also reported on an analysis of viral episodes in pediatric patients with ALL during 2012–2017. A total of 5/251 (2%) children died due to a viral infection (CMV—2 cases, AH1N1—2 cases, and RSV—1 case) [11]. In this previous study, the cumulative incidence was analyzed over two-year periods and we compared these results to those from 2020–2021. The highest frequency of viral infections was observed in the analyzed period of 2020–2021. The cumulative incidence of viral infections in 2020–2021 was over threefold higher than in 2018–2019. This was associated with the emergence of COVID-19 infections, which moreover resulted in the death of two (1%) children. Additionally, in 2020–2021, patients were often admitted to the oncology centers in advanced disease states complicated by infections (viral, bacterial, or fungal) due to fewer visits to primary health providers and the reluctance of families to expose their children to the virus, which delayed the final diagnosis. Moreover, monoclonal antibodies were introduced for the treatment of ALL in Poland at that time, which also increased the frequency of viral infections.

In the other study, the authors presented epidemiology and deaths in children with malignancies and COVID-19 at a reference hospital in Recife, Brazil. A total of 48 children were included, mostly with leukemia (64.5%). The patients had a delay in chemotherapy in the presence of a COVID-19 infection with a median of 15 days in leukemia and four (12.9%) patients that died, mainly during the induction phase of therapy. The authors emphasized that early mortality was less than 2% in cancer patients in the last five years. However, COVID-19 infections increased the death rate more than tenfold [20].

Rojas et al. described 15 pediatric patients with cancer and COVID-19 infections that included 8 children with ALL. Chemotherapy was interrupted in one child but no serious adverse events were observed. The authors reported that the COVID-19 infection rate was 1.3% among this pediatric patients over the first 2 months of the pandemic [21]. 

In the presented study, we observed that COVID-19 infections were predominant in the last two years. In Poland, guidelines related to vaccination children during chemotherapy and after stem cell transplantation have been in place since May 2021. Pediatric patients above the age of 12 years could be vaccinated 3 to 7 days after chemotherapy to minimize severe adverse events from COVID-19 and delays in cancer treatment [22]. 

It is known that viral infections that occur during ALL chemotherapy can cause serious consequences. This is why it is important to reduce the risk of morbidity and mortality via early initiation of antiviral therapy and by educating parents regarding hygiene measures. Education and vaccination (for seasonal influenza, varicella, COVID-19, etc.) of families and medical staff who deal with immunocompromised patients are particularly essential. 

## 5. Conclusions

In conclusion, a high incidence of COVID-19 infections was observed among pediatric patients with ALL in Poland. The mortality rate in our cohort was low (1%). The viral profile in ALL children undergoing chemotherapy can be useful for clinicians to improve prophylactic and therapeutic strategies. 

## Figures and Tables

**Table 1:** Characteristics of patients with ALL who experienced 1 and >1 episode of a viral infection (2020–2021).

Number of Episodes	Total	1	>1	*p*
Number of patients	192	122	70	
Sex, n (%)				
Male	112 (58.3)	66 (54.1)	46 (65.7)	0.156
Female	80 (41.7)	56 (45.9)	24 (34.3)	
Median (Q1; Q3) age at ALL diagnosis, years	4.62 (2.87; 8.94)	4.72 (2.89; 9.37)	4.59 (2.79; 8.50)	0.814
Death related to infection, n (%)	2 (1.0)	1 (0.8)	1 (1.4)	>0.999 ^1^
Infection duration, days				
Median (Q1; Q3)	10.00 (8.00; 13.00)	10.00 (8.00; 11.00)	10.00 (7.00; 14.00)	0.380
Range (days)	0 to 50	0 to 41	2 to 50	

^1^ Dependency between group and sex was analyzed using a chi-squared test and between group and death using Fisher’s exact test. Comparison of age between groups was analyzed using a Mann–Whitney U test. Abbreviations for Table 1: ALL: acute lymphoblastic leukemia.

**Table 2:** The distribution of viral infection episodes and the comparison of frequency of types of viruses between 2018–2019 and 2020–2021.

Type of Viral Infection	Number of Episodes (n = 312 Infections) 2020–2021	Number of Episodes (n = 72) 2018–2019	2020–2021 vs. 2018–2019 ^1^ *p*
Human coronaviruses			
COVID-19	71 (22.8%)	-	<0.001
OC43	10 (3.2%)	-	0.259
HKU1	2 (0.6%)	-	>0.999
Human herpes virus (HHV)			
Cytomegalovirus (CMV, HHV5)	4 (1.3%)	2 (2.8%)	0.693
Epstein–Barr virus (EBV, HHV4)	-	3 (4.2%)	<0.001
Varicella-zoster virus (VZV, HHV3)			0.102
Varicella infection	2 (0.6%)	3 (4.2%)	
Herpes zoster infection	17 (5.4%)	6 (8.3%)	
Herpes simplex virus (HSV-1, HHV1)	14 (4.5%)	-	0.189
Human herpes virus 6 (HHV6)	1 (0.3%)	-	>0.999
Adenovirus (ADV)	16 (5.1%)	35 (48.6%)	<0.001
Rotavirus	15 (4.8%)	23 (31.9%)	<0.001
Norovirus	14 (4.5%)	-	0.138
Human astrovirus (HAstV)	4 (1.3%)		0.748
Sapovirus	2 (0.6%)	-	>0.999
Rhinovirus (HRV)	57 (18.3%)	-	<0.001
Respiratory syncytial virus (RSV)	45 (14.4%)	-	0.001
Human parainfluenza virus (HPIV)			
Type 3	15 (4.8%)	-	0.119
Type 4	11 (3.5%)	-	0.360
Influenza (FLU)			
Type A	3 (1.0%)	-	0.926
Type B	1 (0.3%)	-	>0.999
Human metapneumovirus (MPV)	2 (0.6%)	-	>0.999
BK virus (BKV)	2 (0.6%)	-	>0.999
Parvovirus B19	1 (0.3%)	-	>0.999
Human bocavirus (HBoV)	3 (1.0%)		0.926
Molluscum contagiosum virus (Poxvirus)	1 (0.3%)	-	>0.999

^1^ Data presented as n (% of group from column); comparison of 2 time periods was conducted using a chi-squared test or Fisher’s exact test.

## Data Availability

Not applicable.
